# 

**Published:** 1917-09-29

**Authors:** 


### CONTENTS.

| | PAGE |
|---|---|
| **Efficiency and Individual Freedom** ... ... | 519 |
| **Civilian Practitioners and Military Hospitals** | 520 |
| **Hospital and Institutional News** ... ... ... | 521 |
| Medical V.C.'s—Legacies for Raid-aided Institutions—Further Distributions from the Salomon Estate—Forty-nine Years as a Governor—A Big War-time Extension—The Sights Inspectors do not See—John Wesley and the Hospitals—Letters and Fruit—Poor-Law Infirmaries and Soldiers' Wives—Farm Colony for Tuberculous Sailors and Soldiers—What Causes Measles?—Methods and Measles—The Latest Phase of After-care—Medical Honours—The Germ of Typhus—Malingerer's Jaundice—"Ginger" for Chelmsford—Infant Consultations—No Anxiety about the Plague—The Colour Treatment of Shell-shock—Addison's Disease in a Recruit—An Endowed Bed to the Memory of a Commercial Traveller—Women Porters in Institutions—The Third London *Gazette*—The Helmeted Nurse—This Week's Drug Market—To Our Readers. | |
| **Meat Inspection** ... ... ... ... ... | 527 |
| A Department of Preventive Medicine. | |
| **The After-Care of Consumptives**... .. ... | 528 |
| The Economy of Developing Existing Institutions. | |
| **The Destruction of Medical Certificates** ... | 528 |
| **National Health Insurance Act Developments** | 529 |
| National Insurance and the Local Government Board. | |
| **The Death of Sir Edward Wood**... ... ... | 531 |
| A Notable Hospital Chairman. | |
| **Nursing Affairs in Ireland**... ... ... ... | 532 |
| "Give the College a Chance." | |
| **The Matrons' and Sisters' Department** ... | 533 |
| Matrons in Council: VIII. An All-round View of Reconstruction. | |
| Round the Hospitals. | |
| The Queen and the Patients—Where Princess Louise is Gratefully Appreciated—W.A.A.C.s' Visits to Hospitals—Our Warriors and the Flowers—Flowers as Aids to Recovery—The Ward Tables: A Suggestion. | |
| **Food in Wartime** ... ... ... ... ... ... | 536 |
| The Institution Food Controller. | |
| **The Editor's Letter-Box** ... ... ... ... | 537 |
| C.N.A. Nurses and the "Roll of Merit"—Smoking in Hospital—Sweaters and Other Comforts. | |
| **Passing Events and Topics**... ... ... ... | 537 |
| **Matrons' and Sisters' Appointments** ... ... | 538 |
| **The Medical Schools** ... ... ... ... ... | 538 |
| **Obituary** ... ... ... ... ... ... ... | 538 |
| **Gifts and Bequests** ... ... ... ... ... | 538 |
| **The Institutional Worker** ... ... | (Suppl.) 1 |
| Six Months in Gibraltar. | |
| September Question. | |
| Questions Invited. | |
| Enquiries and Answers. | |
| Employment and Training. | |
| Miscellaneous. | |
| The Housekeepers' Department. | |

